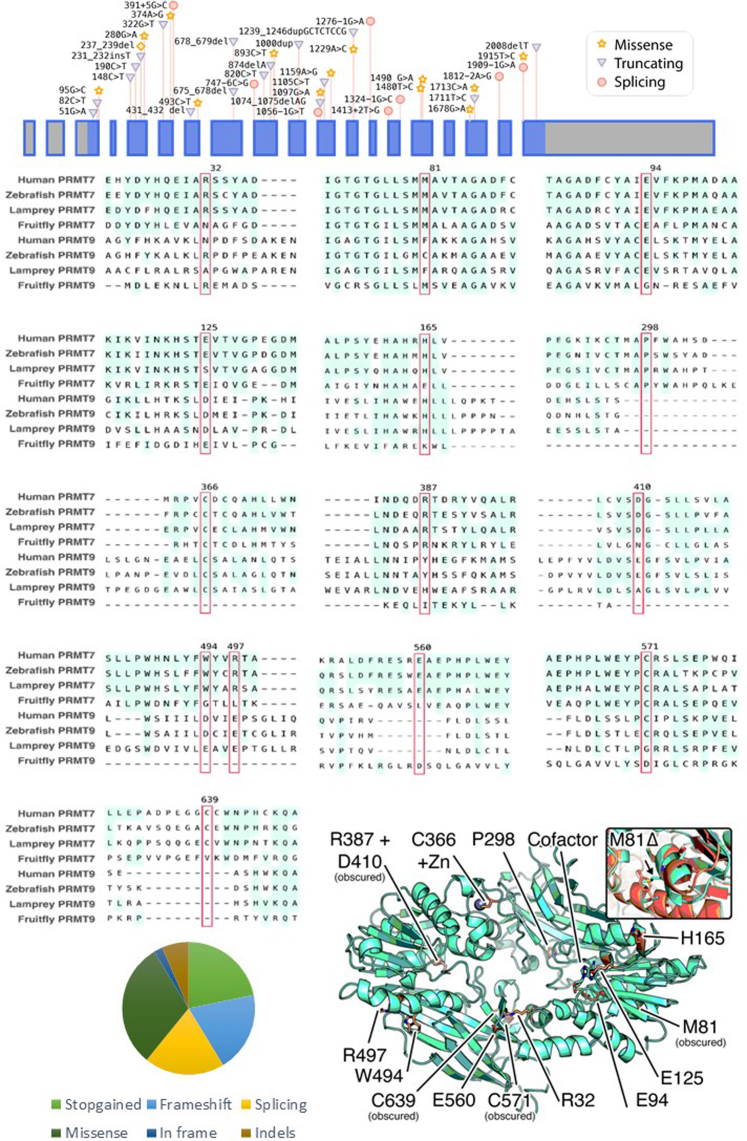# Biallelic *PRMT7* pathogenic variants are associated with a recognizable syndromic neurodevelopmental disorder with short stature, obesity, and craniofacial and digital abnormalities

**DOI:** 10.1016/j.gim.2022.09.016

**Published:** 2023-01

**Authors:** Elisa Cali, Mohnish Suri, Marcello Scala, Matteo P. Ferla, Shahryar Alavi, Eissa Ali Faqeih, Emilia K. Bijlsma, Kristen M. Wigby, Diana Baralle, Mohammad Y.V. Mehrjardi, Jennifer Schwab, Konrad Platzer, Katharina Steindl, Mais Hashem, Marilyn Jones, Dmitriy M. Niyazov, Jennifer Jacober, Rebecca Okashah Littlejohn, Denisa Weis, Neda Zadeh, Lance Rodan, Alice Goldenberg, François Lecoquierre, Marina Dutra-Clarke, Gabriella Horvath, Dana Young, Naama Orenstein, Shahad Bawazeer, Anneke T. Vulto-van Silfhout, Yvan Herenger, Mohammadreza Dehghani, Seyed Mohammad Seyedhassani, Amir Bahreini, Mahya E. Nasab, A. Gulhan Ercan-Sencicek, Zahra Firoozfar, Mojtaba Movahedinia, Stephanie Efthymiou, Pasquale Striano, Ehsan Ghayoor Karimiani, Vincenzo Salpietro, Jenny C. Taylor, Melody Redman, Alexander P.A. Stegmann, Andreas Laner, Ghada Abdel-Salam, Megan Li, Mario Bengala, Amelie Johanna Müller, Maria C. Digilio, Anita Rauch, Murat Gunel, Hannah Titheradge, Daniela N. Schweitzer, Alison Kraus, Irene Valenzuela, Scott D. McLean, Chanika Phornphutkul, Mustafa Salih, Amber Begtrup, Rhonda E. Schnur, Erin Torti, Tobias B. Haack, Carlos E. Prada, Fowzan S. Alkuraya, Henry Houlden, Reza Maroofian

**Affiliations:** 1 Department of Neuromuscular Diseases, UCL Queen Square Institute of Neurology, University College London, London, United Kingdom; 2 Nottingham Clinical Genetics Service, Nottingham University Hospitals NHS Trust, City Hospital Campus, Nottingham, United Kingdom; 3 Department of Neurosciences, Rehabilitation, Ophthalmology, Genetics, Maternal and Child Health (DINOGMI), University of Genoa, Genoa, Italy; 4 Pediatric Neurology and Muscular Diseases Unit, IRCCS Istituto Giannina Gaslini, Genoa, Italy; 5 Genomic Medicine theme, Oxford Biomedical Research Centre, NIHR, Oxford, Oxfordshire, United Kingdom; 6 Wellcome Centre for Human Genetics, Oxford University, Oxford, Oxfordshire, United Kingdom; 7 Department of Cell and Molecular Biology and Microbiology, Faculty of Biological Science and Technology, University of Isfahan, Isfahan, Iran; 8 Palindrome, Isfahan, Iran; 9 Section of Medical Genetics, Children’s Specialist Hospital, King Fahad Medical, City, Riyadh, Saudi Arabia; 10 Department of Clinical Genetics, Leiden University Medical Center, Leiden, The Netherlands; 11 Division of Medical Genetics, Department of Pediatrics, University of California, and Rady Children’s Hospital, San Diego, CA; 12 Wessex Clinical Genetics Service, Princess Anne Hospital, University Hospital Southampton NHS Foundation Trust, Southampton, United Kingdom; 13 Faculty of Medicine, University of Southampton, Southampton General Hospital, Southampton, United Kingdom; 14 Medical Genetics Research Center, Shahid Sadoughi University of Medical Science, Yazd, Iran; 15 Division of Human Genetics, Warren Alpert Medical School of Brown University, Hasbro Children’s Hospital/Rhode Island Hospital, Providence, RI; 16 Institute of Human Genetics, University of Leipzig Medical Center, Leipzig, Germany; 17 Institute of Medical Genetics, University of Zurich, Zurich, Switzerland; 18 Department of Translational Genomics, Center for Genomics Medicine, King Faisal Specialist Hospital and Research Center, Riyadh, Saudi Arabia; 19 Section of Medical Genetics, Department of Pediatrics, Ochsner Health System and University of Queensland, New Orleans, LA; 20 Department of Pediatrics and Molecular and Human Genetics, Baylor College of Medicine, San Antonio, TX; 21 Department of Medical Genetics, Kepler University Hospital Med Campus IV, Johannes Kepler University, Linz, Austria; 22 Children's Hospital of Orange County, Orange, CA; 23 Genetics Center, Orange, California; 24 Department of Neurology, Boston Children’s Hospital, Boston, MA; 25 Division of Genetics and Genomics, Boston Children’s Hospital, Boston, MA; 26 Department of Genetics and Reference Center for Developmental Disorders, Normandie University, UNIROUEN, CHU Rouen, Inserm U1245, FHU G4 Génomique, Rouen, France; 27 Division of Genetics, Department of Pediatrics, David Geffen School of Medicine, University of California at Los Angeles, Los Angeles, CA; 28 BC Children’s Hospital Research Institute, BC Children’s Hospital, Vancouver, British Columbia, Canada; 29 Department of Pediatrics, University of British Columbia, Vancouver, Canada; 30 Adult Metabolic Diseases Clinic, Vancouver General Hospital, Vancouver, Canada; 31 Pediatric Genetics Unit, Schneider Children’s Medical Center of Israel, Petach Tikva, Israel; 32 Sackler Faculty of Medicine, Tel Aviv University, Tel Aviv, Israel; 33 Department of Human Genetics, Radboud University Medical Center, Nijmegen, The Netherlands; 34 Genetica AG, Zürich, Switzerland; 35 Dr. Seyedhassani Medical Genetic Center, Yazd, Iran; 36 KaryoGen, Isfahan, Iran; 37 Department of Human Genetics, Graduate School of Public Health, University of Pittsburgh, PA; 38 Department of Neurosurgery, Program on Neurogenetics, Yale School of Medicine, Yale University, New Haven, CT; 39 Masonic Medical Research Institute, Utica, NY; 40 Children Growth Disorder Research Center, Shahid Sadoughi University of Medical Sciences, Yazd, Iran; 41 Department of Medical Genetics, Next Generation Genetic Polyclinic, Mashhad, Iran; 42 Molecular and Clinical Sciences Institute, St. George's, University of London, London, United Kingdom; 43 Innovative Medical Research Center, Mashhad Branch, Islamic Azad University, Mashhad, Iran; 44 Yorkshire Regional Genetics Service, Chapel Allerton Hospital, Leeds, United Kingdom; 45 Department of Clinical Genetics, Maastricht University Medical Center, Maastricht, The Netherlands; 46 MGZ - Medizinisch Genetisches Zentrum, Munich, Germany; 47 Human Genetics and Genome Research Division, Department of Clinical Genetics, National Research Centre, Cairo, Egypt; 48 Invitae, San Francisco, CA; 49 Laboratory of Medical Genetics, Tor Vergata Hospital, Rome, Italy; 50 Autophagy Laboratory, Department of Molecular Biology, Interfaculty Institute of Cell Biology, Eberhard Karls University Tuebingen, Tuebingen, Germany; 51 Medical Genetics Department, Bambino Gesù Children's Hospital, IRCCS, Rome, Italy; 52 Genetics and Rare Diseases Research Division, Bambino Gesù Children’s Hospital, IRCCS, Rome, Italy; 53 West Midlands Regional Genetics Service and Birmingham Health Partners, Birmingham, United Kingdom; 54 Women's and Children’s NHS Trust, Birmingham, United Kingdom; 55 Castle Hill Hospital, Cottingham, Hull, United Kingdom; 56 Department of Clinical and Molecular Genetics, University Hospital Vall d'Hebron, Barcelona, Spain; 57 Medicine Genetics Group, Valle Hebron Research Institute, Barcelona, Spain; 58 Division of Pediatric Neurology, Department of Pediatrics, College of Medicine, King Saud University, Riyadh, Saudi Arabia; 59 Department of Pediatrics, College of Medicine, AlMughtaribeen University, Khartoum, Sudan; 60 GeneDx, Gaithersburg, MD; 61 Institute of Human Genetics and Applied Genomics University of Tübingen, Tübingen, Germany; 62 Centre for Rare Diseases, University of Tübingen, Tübingen, Germany; 63 Department of Pediatrics, Cincinnati Children’s Hospital, Cincinnati, OH; 64 Division of Genetics, Birth Defects and Metabolism, Ann & Robert H. Lurie Children’s Hospital of Chicago, Chicago, IL; 65 Department of Pediatrics, Feinberg School of Medicine of Northwestern University, Chicago, IL; 66 Department of Anatomy and Cell Biology College of Medicine, Alfaisal University, Riyadh, Saudi Arabia

**Keywords:** Chromatinopathy, Mendelian disorders of the epigenetic machinery, PRMT7, Syndromic neurodevelopmental disorder, Syndromic obesity

## Abstract

**Purpose:**

Protein arginine methyltransferase 7 (PRMT7) is a member of a family of enzymes that catalyzes the methylation of arginine residues on several protein substrates. Biallelic pathogenic *PRMT7* variants have previously been associated with a syndromic neurodevelopmental disorder characterized by short stature, brachydactyly, intellectual developmental disability, and seizures. To our knowledge, no comprehensive study describes the detailed clinical characteristics of this syndrome. Thus, we aim to delineate the phenotypic spectrum of *PRMT7*-related disorder.

**Methods:**

We assembled a cohort of 51 affected individuals from 39 different families, gathering clinical information from 36 newly described affected individuals and reviewing data of 15 individuals from the literature.

**Results:**

The main clinical characteristics of the *PRMT7*-related syndrome are short stature, mild to severe developmental delay/intellectual disability, hypotonia, brachydactyly, and distinct facial morphology, including bifrontal narrowing, prominent supraorbital ridges, sparse eyebrows, short nose with full/broad nasal tip, thin upper lip, full and everted lower lip, and a prominent or squared-off jaw. Additional variable findings include seizures, obesity, nonspecific magnetic resonance imaging abnormalities, eye abnormalities (i.e., strabismus or nystagmus), and hearing loss.

**Conclusion:**

This study further delineates and expands the molecular, phenotypic spectrum and natural history of *PRMT7*-related syndrome characterized by a neurodevelopmental disorder with skeletal, growth, and endocrine abnormalities.

## Graphical abstract

**Figure:**
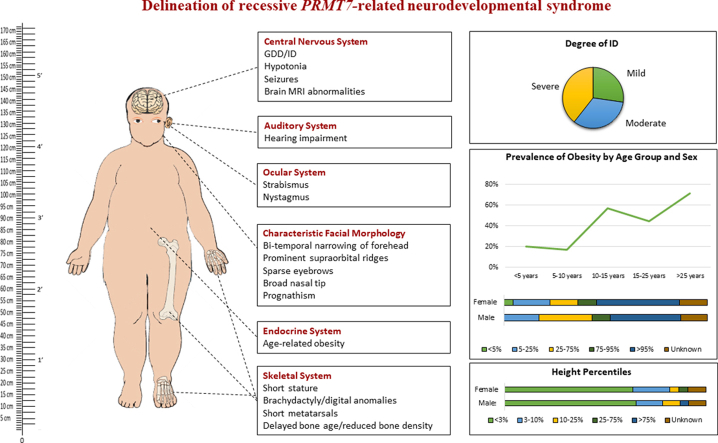

## Introduction

Protein arginine methyltransferase 7 (PRMT7) is a member of a family of enzymes that catalyze the methylation of arginine residues on several protein substrates. Arginine methylation plays a crucial role in various biological pathways, influencing chromatin, RNA biology, and phase separation.^1^^,^^2^ The PRMT family regulates physiological functions, and its dysfunction is linked to pathologies as diverse as cancer, inflammation, and neurodegeneration. PRMT7 is a unique, evolutionarily conserved PRMT family member that catalyzes the monomethylation of arginine.^3^ Biallelic pathogenic variants in *PRMT7* (OMIM ∗ 610087) have been associated with a syndromic neurodevelopmental disorder characterized by short stature, brachydactyly, intellectual developmental disability, and seizures, which is currently known as SBIDDS syndrome^4^, ^5^, ^6^, ^7^, ^8^, ^9^ (OMIM 617157). Since the identification of *PRMT7* as a disease-associated gene in 2015, 15 cases from 9 families have been published in 6 separate reports. Until now, to our knowledge, there have been no comprehensive studies describing the characteristics and phenotypic spectrum of the *PRMT7*-related disorder.

Here, we report a large cohort of 51 affected individuals from 39 different families, assembled by gathering clinical information from 36 newly described patients and reviewing data of 15 affected individuals from the literature.

## Materials and Methods

The affected individuals were identified through data sharing with collaborators and screening the databases of several diagnostic and research genetic laboratories worldwide, as well as using GeneMatcher.^10^ Next-generation sequencing was performed on genomic DNA extracted from blood in different diagnostic or research laboratories worldwide. The candidate variants were confirmed, and segregation analysis was performed by Sanger sequencing. Detailed clinical data and family history were collected for new and published cases in the form of completing a clinical proforma by the recruiting clinicians. Detailed assessment of facial morphology was performed on clinical photographs by a clinical geneticist expert in dysmorphology (M.Su.). Where permission to share clinical photographs was not given, information on the dysmorphological features was provided by clinical assessment performed by the family’s clinicians.

## Results

### Clinical characterization

**Figure 1:**
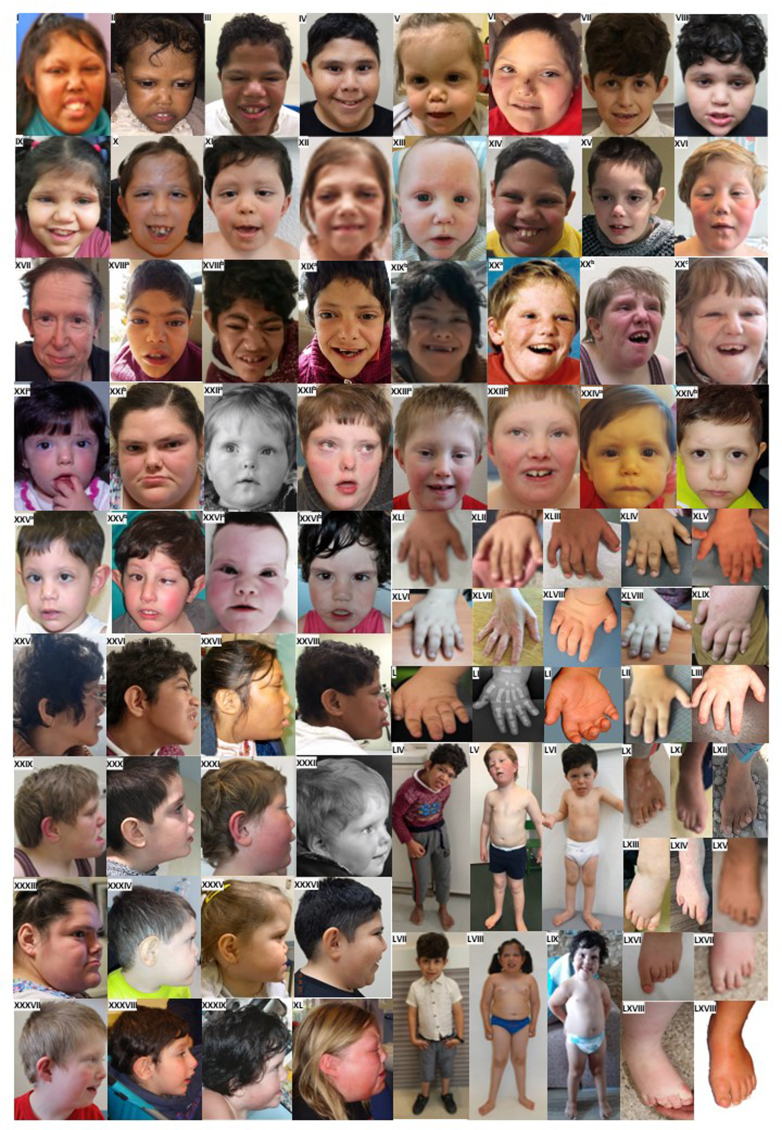
**Phenotypic presentation of individuals with *PRMT7*-related syndrome.** From I to XVI: craniofacial features of affected individuals, front view. From XVII^a^ to XXIV^d^: evolution of facial features among different ages. From XXV to XL: craniofacial features of affected individuals, sagittal view. From XLI to LIII: digital abnormalities. From LIV to LIX: stature of affected individuals. From LX to LXVIII: foot abnormalities.

**Figure 2:**
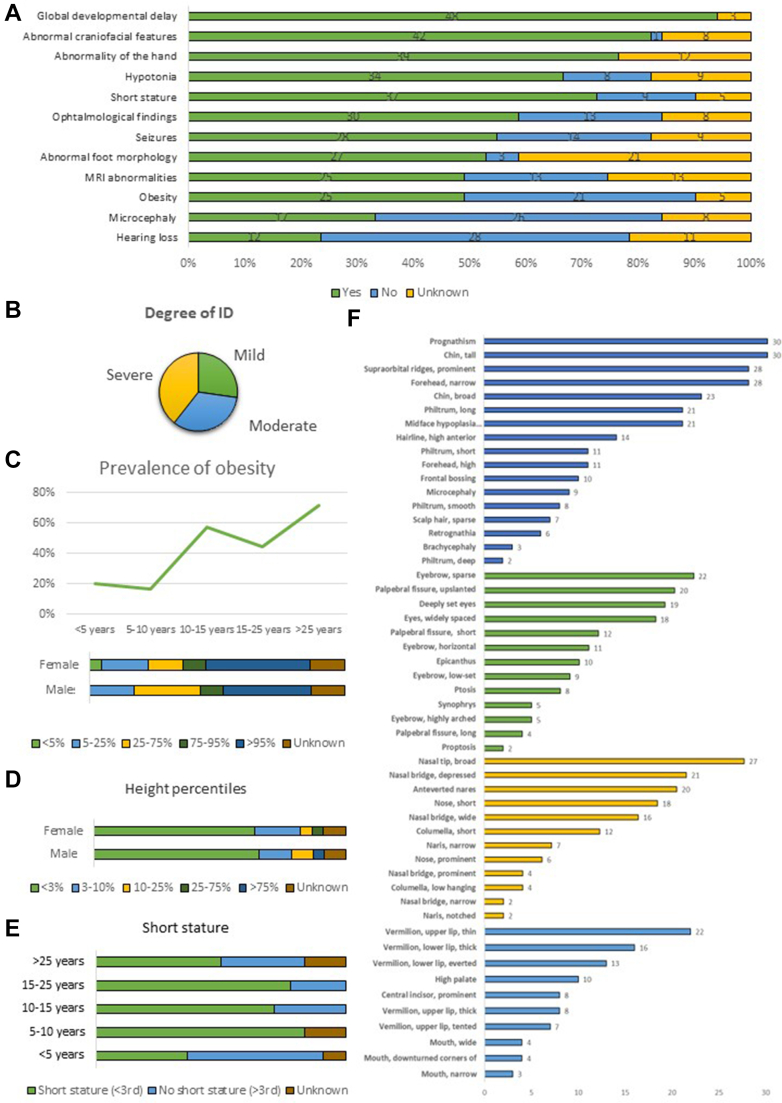
**Overview of the main clinical features of *PRMT7*-related disorder.** A. Occurrence of the main phenotypic features in the cohort. Green: Phenotype present. Blue: Phenotype not present. Yellow: Information not available. B. Degree of intellectual disability. C. Prevalence of obesity by age group and sex. D. Distribution of height percentile throughout the cohort. E. Prevalence of short stature among different ages. F. Deep characterization of the main dysmorphological features of the craniofacial (blue), periorbital (green), nasal (yellow), and perioral (light blue) areas.

The cohort consisted of 23 males and 28 females whose age at last evaluation ranged between 34 weeks of gestation to 55 years (median 9.5 years, interquartile range 5.4-17.8 years). Consanguinity was reported in 16 families (16 of 36, 44%). Figure 1 displays the facial appearance of individuals for whom photographs were available. Figure 2 gives an overview of the core clinical findings, summarized in Supplemental Table 1.

Most individuals were born at term. Pregnancy was uneventful with unremarkable perinatal period for 20 individuals (20 of 43, 45%), whereas 23 individuals (22 of 40, 55%) presented with intrauterine growth restriction (14 of 31, 45%) and/or other variable prenatal manifestations (20 of 41, 48%). Nineteen newborns were described as small for gestational age (19 of 41, 46%). Weight at birth was within the normal range for 20 children, less than the 5th percentile for 19 children, and greater than the 95th percentile for 1 child. Decreased head circumference at birth was described in 6 children (6 of 23, 26%). At last evaluation, microcephaly was reported in 17 individuals (17 of 43, 40%). When specified, failure to thrive was documented in 18 individuals (18 of 35, 51%), and most individuals presented short stature (37 of 46, 80%). If available, findings from x-ray imaging included decreased bone density (4 of 10, 40%) and/or delayed bone age (5 of 10, 50%). Twenty-five affected individuals were either overweight (*n* = 5) or obese (*n* = 20) at last evaluation (25 of 46, 54%). Interestingly, obesity was more commonly reported in individuals older than 10 years, suggesting a delayed onset.

Notably, all individuals (48 of 48, 100%) showed global developmental delay (GDD) in early infancy and intellectual disability (ID). Where specified (*n* = 33), the degree of ID was mild in 9 (27%), moderate in 11 (33%), and severe in 13 (40%). Hypotonia was reported in most individuals (37 of 42, 88%, where specified *n* = 10 generalized, *n* = 5 axial), often manifesting in the neonatal period. Seizures developed in 28 individuals (28 of 42, 67%) with a median age of seizure onset of 3 years (range: 7 months-45 years, interquartile range: 1-4.8). Seizure onset varied from generalized (*n* = 18) to focal (*n* = 4). Generalized-onset seizures include tonic-clonic (*n* = 9), absence (*n* = 6), atonic (*n* = 5), and myoclonic (*n* = 2) seizures. Febrile seizures were reported in 2 individuals. Only 4 patients (4 of 25, 16%) presented with intractable seizures, whereas most showed good response to treatments (ie, topiramate, sodium valproate) or were seizure-free without therapy. Some individuals presented with strabismus (19 of 42, 45%) and hearing impairment (12 of 40, 30%). Of the 37 brain magnetic resonance imaging scans for which a clinical report was available, 13 were described as unremarkable. Magnetic resonance imaging findings included nonspecific changes, such as enlargement of the ventricles or subarachnoid spaces, white matter abnormalities, and mild parenchymal atrophy.

### Dysmorphology assessment

Photographs were available from 28 new affected individuals from 23 families. These included childhood and adult photographs from 3 patients, including 2 siblings from 1 family (patients 6, 7, and 8). Facial features of 12 previously reported patients were also reviewed.^4^, ^5^, ^6^, ^7^^,^^9^ These included 3 adults, 1 adolescent, and 8 children. Photographs of the hand(s) and feet were only available for 19 new affected individuals from 15 families and 10 previously reported patients from 7 families.^4^, ^5^, ^6^, ^7^^,^^9^ There was no consent to share photographs of individuals 13, 15, 19, 20, 29, 30, and 36, and information on their dysmorphological features was retrieved from the clinical assessment performed by their clinicians.

Based on the available photographs, the most recurrent facial dysmorphic features were frontal bossing/prominent supraorbital ridges (70%), bitemporal (bifrontal) narrowing of forehead (67.5%), prognathism (72.5%), sparse eyebrows (55%), broad nasal tip (62.5%), and tall (prominent) chin (75%). Less frequently seen features included mid-face hypoplasia (47.5%), deep-set and widely spaced eyes (42.5%), upslanted palpebral fissures (45%), short nose (40%), depressed nasal bridge (47.5%), short columella (30%), long philtrum (45%), thin upper lip vermilion (47.5%), thick lower lip vermilion (40%), everted lower lip vermilion (32.5%), and a broad chin (52.5%). Another commonly reported feature was short neck. Many affected individuals also had changes affecting their fingers and toes. These included short fingers (brachydactyly) (70%), broad thumbs (37.5%), and short toes or short metatarsals (45.9%). The characteristic facial dysmorphism is not seen in very young children but can be seen in older children and adults. Detailed dysmorphological features of each individual can be found in Supplemental Table 2.

### *PRMT7* variants

A total of 51 individuals with either compound heterozygous (*n* = 25, biallelic inheritance from unaffected parents) or homozygous (*n* = 26, biallelic inheritance from unaffected parents) *PRMT7* variants were included in this cohort. Forty-six variants were detected, of which 34 were not previously reported in the literature. This includes 19 truncating variants (*n* = 9 frameshift, *n* = 10 nonsense), 1 in-frame deletion, 9 splicing, 14 missense variants, and 3 large indels. All identified variants are absent or found at very low allele frequencies in several variant databases (range 0.0–0.00002), are predicted to be damaging across a suite of in silico tools, and affect highly evolutionarily conserved residues. According to the American College of Medical Genetics and Genomics and the Association for Molecular Pathology classification,^11^^,^^12^ 28 variants were classified as pathogenic, 3 variants as likely pathogenic, and 12 variants were of uncertain significance. Variants of uncertain significance were then reclassified as likely pathogenic/pathogenic based on striking similarity to other cases and results of co-segregation studies. The characteristics of the variants are summarized in Supplemental Table 3 and shown in Supplemental Figure 1 and interactively at https://michelanglo.sgc.ox.ac.uk/r/prmt7

## Discussion

We report on the molecular and phenotypic spectrum of 51 individuals with biallelic *PRMT7* variants, representing the most comprehensive study to date. The phenotypic spectrum of *PMRT7*-related disorder includes GDD/ID, short stature, distinct craniofacial and digital defects, epilepsy, and obesity.

PRMTs are a family of enzymes that catalyze the methylation of arginine residues on several protein substrates. Arginine methylation has been largely studied for its key role in post-translational modification, influencing several biological processes, such as messenger RNA splicing, DNA repair, transcription regulation, and signal transduction.^1^^,^^2^ Known substrates methylated by *PRMT7* include core histones (H2A, H2B, H3, and H4), Wnt signaling molecules, and transcription factors, involving *PRMT7* in a wide range of biological processes, such as gene expression and epigenetic regulation, cell differentiation, muscle and neuron development, and adipogenesis.^3^^,^^13^^,^^14^ A significant number of genes involved in chromatin regulation have been associated with neurodevelopmental disorders, collectively known as chromatinopathies.^15^ *PRMT7-*related disorder shares some overlapping phenotypes with chromatin-related neurodevelopmental disorders, particularly short stature and growth delay and presence of major craniofacial dysmorphisms and skeletal and digital abnormalities (eg, Wiedemann-Steiner syndrome and Chung-Jansen syndrome). Given its known importance in histone methylation and genome regulation, *PRMT7*-related syndrome could potentially be considered as one of the Mendelian disorders of the epigenetic machinery.^16^

As further confirmation of its crucial function, *PRMT7* has hitherto been the sole member of this family to be associated with a monogenic disorder. *Prmt7*-deficient mice generated by Jeong et al^17^ in 2016 exhibited defects in bone and skeletal muscle mass, delayed or impaired neuronal development, and increased adipogenesis, resembling many aspects of the human phenotype.

*PRMT7* plays an important role in neuronal development and differentiation, caused by interaction with MLL4^18^ and Wnt signaling molecules, and in HCN (hyperpolarization-activated, cyclic-nucleotide-gated) channel functioning and neuronal excitability, through regulation of SHANK3 and NALCN, respectively.^19^ Consistent with such a role, all the affected individuals in our cohort presented with GDD/ID, and 70% of them developed seizures.

Although *PRMT7*’s role in bone remodeling and development is still uncertain, adult mutated mice showed limb bone anomalies and reduced length. Likewise, 80% of the affected individuals of our cohort were diagnosed with short stature and, if available, findings from x-ray imaging included decreased bone density or delayed bone age and metacarpal and metatarsal shortening.

There is increasing evidence of the pivotal role of *PRMT7* in adipogenesis and muscle development. *PRMT7* negatively regulates adipocyte differentiation through modulation of *C/EBP-β* and therefore *PPAR-γ2*.^20^ *Prmt7* knockout mice showed decreased energy expenditure and developed age-related obesity with excessive body fat accumulation at middle age. Similarly, half of our cohort exhibited obesity, with a higher prevalence after puberty. Half of the newborns were small for gestational age, consistent with the finding that the *Prmt7*-deficient mice displayed reduced body size and weight at birth. The same mice showed a switch from oxidative to glycolytic fibers in muscle before obesity development, suggesting that the consequent imbalance in energy homeostasis might be the cause of the obesity in mice and implying *PRMT7* as an important co-factor in adult muscle development. Similarly, some of our patients reported reduced endurance exercise capacities. We hereby confirm the importance of *PRMT7* function in human adipogenesis, suggesting that it might be a potential target for intervention and treatment of obesity. We suggest that *PRMT7-*related disorder should be considered in the differential diagnosis of monogenic syndromic obesity. Syndromic forms of obesity that may share overlapping features with *PRMT7*-related disorder include Borjeson-Forssman-Lehmann syndrome, CHOPS syndrome, Chung-Jansen syndrome, Cohen syndrome, and *TRAPPC9*-related disorder.^21^

After performing systematic review of the facial features of our cohort, we observed that *PRMT7*-related disorder shows a recognizable facial gestalt that is distinguishable from the other conditions mentioned above. This disorder should be suspected, in the context of a likely recessive inheritance, in patients with DD/ID who have digital abnormalities, bifrontal narrowing, prominent supraorbital ridges, sparse eyebrows, short nose with full/broad nasal tip, thin upper lip, full and everted lower lip, and a prominent or squared-off jaw. Some other important points in the differential diagnosis have been discussed and included in Supplemental Table 4.

There is also a considerable overlap with the phenotype associated with pseudohypoparathyroidism type 1A and 1C (Albright hereditary osteodystrophy), acrodysostosis, and chromosome 2q37 deletion syndrome (including brachydactyly-mental retardation syndrome because of loss-of-function variants in the *HDAC4*). This includes not only DD/ID but also short stature, similar facial dysmorphic features, short fingers and toes with broad thumbs, metacarpal and/or metatarsal shortening, and the tendency to excessive weight gain. However, unlike PRMT7-related disorder, these are all autosomal dominant disorders caused by heterozygous variants in *GNAS* (PHP 1A and 1C), *PRKAR1A* and *PDE4D* (acrodysostosis), and/or 2q37 deletion resulting in loss of the *HDAC4.* Furthermore, patients with these disorders can have normal neurodevelopment and can show evidence of multiple hormone resistance.^22^

In conclusion, we delineate and expand the phenotypic spectrum and natural history of the disease associated with pathogenic biallelic *PRMT7* variants, providing a comprehensive review of the associated clinical phenotype. In addition, we characterize the distinct craniofacial morphology of this syndrome. This study provides a valuable resource for clinicians for the accurate diagnosis, assessment, counseling, and better management of affected individuals with this rare syndrome. Further studies will be needed to deeply understand *PRMT7* regulatory functions and possibly identify strategies for therapy.

## Data Availability

The authors declare that the data supporting the findings of this study are available within the paper and its supplementary information files.

## Conflict of Interest

Megan Li is an employee of Invitae. Erin Torti, Amber Begtrup, and Rhonda E Schnur are employees of GeneDx, Inc. All other authors declare no conflicts of interest.

## Figures and Tables

**Supplementary Figure S1:**