# Supplementary material for: Biallelic PRMT7 pathogenic variants are associated with a recognizable syndromic neurodevelopmental disorder with short stature, obesity, and craniofacial and digital abnormalities
Source: Genet Med. 2023 Jan;25(1):135–42. doi: 10.1016/j.gim.2022.09.016 (PMC10620944; doi:10.1016/j.gim.2022.09.016)
Supplement: Supplementary Table S4 [file mmc4.docx]

| Disorder | Group | OMIM number | Gene | Mode of Inheritance | Distinguishable facial dysmorphisms | GDD/ID | Seizures | Hypotonia | Short stature | Obesity | Digital abnormalities | Other differential features |
| --- | --- | --- | --- | --- | --- | --- | --- | --- | --- | --- | --- | --- |
| PRMT7-related disorder (short stature, brachydactyly, intellectual developmental disability, and seizures) | Chromatinopathy/  syndromic obesity | 617157 | *PRMT7* | AR | Bi-frontal narrowing, prominent supraorbital ridges, sparse eyebrows, short nose with full/broad nasal tip, thin upper lip, full and everted lower lip and a prominent or squared-off jaw | + | + | + | + | + | + |  |
| Acrodysostosis 1 and 2 | Impaired G protein-coupled receptor signaling | 101800  614613 | *PRKAR1A*  *PDE4D* | AD | Flat anterior facies, mid-face hypoplasia, hypertelorism, small nose, depressed nasal bridge, short columella, long philtrum, prominent chin | ± | - | - | + | + | + | Severe brachydactyly, skeletal dysplasia, multiple hormone resistance |
| Borjeson-Forssman-Lehmann (BFL) syndrome | Syndromic Obesity | 301900 | *PHF6* | XLR | Deep-set eyes, large ears with large lobes | + | + | + | + | + | + | Tapering fingers, and rather hypoplastic external genitalia with pubertal delay |
| CHOPS syndrome | Syndromic Obesity | 616368 | *AFF4* | AD | Arched eyebrows, synophrys, long eyelashes, and upturned nasal tip in younger patients and coarsening of the face in older children | + | - | - | + | + | + | Heart anomalies (patent ductus arteriosus and ventricular septal defect), pulmonary and respiratory tract involvement, and skeletal dysplasia with abnormal vertebral shape |
| Chromosome 2q37 deletion/brachydactyly-mental retardation syndrome | Chromatinopathy | 600430 | *HDAC4* | AD | Round face, frontal bossing, deep-set eyes, up-slanting palpebral fissures, broad nose, depressed nasal bridge, anteverted nares, thin upper lip | ± | ± | - | + | + | + | Self-injurious behaviour, sleeping difficulties, congenital heart anomalies, x-ray features of brachydactyly type E |
| Chung-Jansen Syndrome | Chromatinopathy/  Syndromic obesity | 617991 | *PHIP* | AD | Large ears, thick eyebrows, synophrys, small nose | + | - | + | - | + | + | Tapering fingers, Cafe au lait spots, behavioral manifestations |
| Cohen syndrome | Syndromic Obesity | 216550 | VPS13B | AR | Bushy eyebrows and eyelashes, down-slanting palpebral fissures with a wave-shaped outline, high nasal bridge, low-set columella, and a short, upturned philtrum with prominent central incisors | + | + | + | + | + | + | High myopia and retinal dystrophy, narrow hands with slender fingers, narrow feet with sandal gap, pubertal delay and neutropenia |
| Pseudohypoparathyroidism  Type IA and IC  (Albright hereditary osteodystrophy) | Impaired G protein-coupled receptor signaling | 103580  612462 | *GNAS* | AD | Round face, full cheeks, depressed nasal bridge | ± | + | - | + | + | + | Multiple hormone resistance, subcutaneous calcification |
| TRAPPC9-related disorder (mental retardation, autosomal recessive 13) | Syndromic Obesity | 613192 | *TRAPPC9* | AR | Hypoplastic supraorbital ridges | + | + | + | - | + | + | Tapering fingers, stereotypies, MRI changes (cerebral and cerebellar atrophy, thin corpus callosum, and multifocal supratentorial white matter abnormalities) |
| Wiedemann-Steiner syndrome | Chromatinopathy | 605130 | *KMT2A* | AD | Thick eyebrows, synophrys and thick eyelashes with vertically narrow and down-slanting palpebral fissures. | + | + | + | + | - | + | Hypertrichosis over their forearms, legs and back |

**Supplementary Table S4. Overview of disease genes which are of interest in the differential diagnosis of PRMT7-associated neurodevelopmental disorder.**

**Supplementary Figure 1.**

a) Schematic representation of the *PRMT7* gene with all variants reported, including those reported in the present study. b) Alignment of human PRMT7 and PRMT9 protein sequence surrounding the detected missense variants with other model organisms including Zebrafish, Lamprey and Fruitfly. c) Locations of the residues affected in the various variants. The represented structure of PRMT7 is a homology model and features ligands from its template, namely a coordinated zinc and S-adenosyl methionine, whose binding is predicted to be compromised in the Cys366Tyr and Glu94Lys variants respectively. The in-frame deletion of Met81 disrupts the turns of the C-terminal end of helix (insert, wild type: turquoise, in-frame deletion in salmon) and despite nearby residues predicted to repack to accommodate this, such as Tyr49 packing where Met81 is in the wild type (arrow), the variant is predicted to be highly destabilised (+27 kcal/mol). d) Schematic illustration of the type of variants reported in *PRMT7*.
